# Dietary diversity, meal frequency and associated factors among infant and young children in Northwest Ethiopia: a cross- sectional study

**DOI:** 10.1186/s12889-015-2333-x

**Published:** 2015-10-03

**Authors:** Melkamu Beyene, Abebaw Gebeyehu Worku, Molla Mesele Wassie

**Affiliations:** Department of Public Health, Mizan-Tepi University, P.O. Box 260, Mizan-Aman, Ethiopia; Department of Reproductive Health, Institute of Public Health, University of Gondar, P.O. Box 196, Gondar, Ethiopia; Department of Human Nutrition, Institute of Public Health, University of Gondar, P.O. Box 196, Gondar, Ethiopia

**Keywords:** Dietary diversity, Meal Frequency, Infant and young child, Ethiopia

## Abstract

**Background:**

Inappropriate feeding practice increases risk of under nutrition, illness, and mortality amongst children less than 2 years of age. The objective of this study is to assess minimum dietary diversity, meal frequency and its associated factors among infant and young children aged 6–23 months in Dangila Town, Northwest Ethiopia.

**Methods:**

A community based cross sectional study was conducted. Simple random sampling technique was used to select study participants. Interviewer administered questionnaire were used. Bivariate and multivariable logistic regression analyses was employed to identify factors associated with minimum dietary diversity and meal frequency.

**Results:**

A total of 920 children 6–23 months were included. Proportion of children who met the minimum dietary diversity and meal frequency was 12.6 and 50.4 %, respectively. Mothers education [AOR =2.52], age of a child [AOR = 2.05], birth order of index child [AOR = 2.08], living in urban area [AOR = 2.09], having home gardening [AOR = 2.03], and media exposure [AOR = 2.74] were positively associated with dietary diversity. Moreover, age of the child [AOR = 3.03], birth order of index child [AOR = 1.58], mothers involvement in decision making [AOR = 1.51], media exposure [AOR = 2.62], and having postnatal visit [AOR = 2.30] were positively associated with meal frequency.

**Conclusion:**

The proportion of children who received minimum dietary diversity and meal frequency was low. Being at younger age, first birth order, and lack of media exposure affect both dietary diversity and meal frequency. Increasing mother’s education, home gardening, mass media promotion and empowering women in decision making are highly recommended to increase dietary diversity and meal frequency.

## Background

Proper infant and young child feeding practice is needed in the first two years of life for optimal child growth, better health, and development. Complementary feeding practice is a process of starting other foods besides breast milk to meet the increasing demand in terms of nutritional requirement [1–3].

**Fig. 1:**
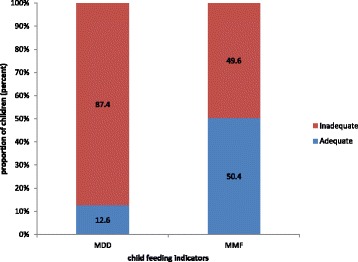
Proportion of children received recommended minimum dietary diversity and meal frequency practices among 06–23 months of children, Dangila, Northwest Ethiopia, 2014. *MDD* minimum dietary diversity, *MMF* minimum meal frequency

According to the World Health Organization (WHO), indicators of proper complimentary feeding are starting of solid, semi-solid or soft foods, minimum meal frequency, minimum dietary diversity, minimum acceptable diet, and consumption of iron-rich or iron-fortified foods [4].

Inappropriate complementary feeding practices increase the risk of under nutrition, illness, and mortality in infants and young children less than 2 years of age [5, 6]. Greater than two-thirds of malnutrition related child deaths are associated with inappropriate feeding practices during the first two years of life in such a way that infants and young children received inadequately nutritious diets, poorly diversified and infrequently feeding [7]. Malnourished children who survive are also getting more frequently sick and suffer from life-long consequences of malnutrition in their life and perhaps the effect will span generation [6, 7].

In Ethiopia, 40 % of children are stunted, 9 % are wasted, and 25 % are underweight which informed the occurrence of both acute and chronic under nutrition. Only 4 % of children have been fed using minimum acceptable diet [8]. Detailed analysis of EDHS 2011 showed that 10.8 and 44.7 % of children aged 6–23 months have received minimum dietary diversity and minimum meal frequency, respectively in Ethiopia [9].

Hence the prevalence of malnutrition is still high among the children in Ethiopia and malnutrition is highly associated with low complementary feeding practices (dietary diversity and meal frequency), it is imperative that further research is essential to find out actual feeding practices and factors associated with dietary diversity and meal frequency among Infant and young children.

## Methods

### Study design, area and period

A community based cross-sectional study was conducted to assess the prevalence of minimum dietary diversity and meal frequency among infant and young children aged 6 to 23 months. This study was conducted at Dangila town from March 1–30, 2014. Dangila is located at a distance of 476 km from Addis Ababa (capital city of Ethiopia) in the Northwest direction.

### Source population and sampling

The source population was all infant and young children 6–23 months old who lived in Dangila. For sample size calculation we used single population proportion formula considering the following assumptions: 95 % confidence level, Proportion (P) of 10.8 % for minimum dietary diversity and 44.7 % for minimum meal frequency and margin of error of 2 % for minimum dietary diversity (rare event) and 5 % for minimum meal frequency. N_1_ for minimum diet diversity and N_2_ for minimum meal frequency: − n_1_ = (1.96)^2^*(0.108)*(1–0.108)/(0.02)^2^ = 925 and n_2_ = (1.96)^2^*(0.447)*(1–0.447)/(0.05)^2^ = 380. However, for having larger power, we have taken n = 925 for our final sample size. . Lists of infants and young children aged between 6 and 23 months along with their mothers residing in all kebeles (the smallest administrative unit) of Dangila Town were taken from health extension workers and then sampling frame was constructed for each kebeles. Simple random sampling technique was used to select a sample of 925 children proportionally from all kebeles by using lottery method.

### Data collection procedures

Data were collected using interviewer- administered and structured questionnaire. The data collection tool regarding the various factors was adopted from EDHS 2011 questionnaire with some modification to fit with the context Moreover, data on dietary diversity and meal frequency were adopted from WHO standardized questionnaire for IYCF practices. This was based on the mother’s recall of foods given to her child in the past twenty four hours (24 h) before the survey.

Household wealth was constructed using principal components analysis to determine the weights for the wealth based on information collected about several household assets and facilities. This wealth index was divided into three categories as poor, middle and rich and each household was assigned to one of these categories.

### Operational definitions

#### Minimum dietary diversity

Proportion of children with 6–23 months of age who received foods from four or more food groups of the seven food groups. The seven foods groups used for tabulation of this indicator were: grains, roots and tubers; legumes and nuts; dairy products (milk, yogurt); Flesh foods (meat, fish, poultry and liver/organ meats); eggs; vitamin A-rich fruits and vegetables; and other fruits and vegetables. Consumption of any amount and quality of food from each food group was sufficient to ‘count’, *i.e.,* there was no minimum quantity, except if an item was only used as a condiment [4].

#### Minimum meal frequency

Proportion of breastfed and nonbreastfed children aged 6–23 months who received solid, semisolid, or soft foods (but also including milk for non-breastfed children). The Minimum frequency was defined as: twice for breastfed infants 6–8 months, three times for breastfed children 9–23 months, and four times for non-breastfed children 6–23 months [4].

#### Satisfactory exposure to media

Women aged 15–49 years at least once a week read a newspaper or magazine or listen to radio, or watched television.

### Data processing and analysis

Data were entered and cleaned using EPI-info version 3.5.3 statistical software and then exported to SPSS version 20.0 statistical software for analysis. Dietary diversity and meal frequency variables were dichotomized as category 0 for not meeting the minimum criteria and otherwise taken as category 1. Descriptive statistics were done. Bivariate logistic regression analysis was used to assess the association between each independent variable with the dependent variables. Those variables that have been associated with the dependent variables at p-value of less than 0.2 were fitted in to multivariate logistic regression models to control the effects of confounding. Those variables having p- value of less than 0.05 was considered as significant.

### Ethical considerations

Ethical clearance was obtained from the Ethical Review Board of the Institute of Public Health of university of Gondar. Verbal consent was obtained from the participant’s mother after informing them all the purpose, benefit, risk, the confidentiality of the information and the voluntary nature of participation in the study. Participants found to have mal practices regarding their infant or young child feeding had been counseled after the completion of the interview.

## Results and discussion

### Characteristics of the sample

A total of 920 infants and young children aged 6 to 23 months along with their mothers were enrolled in the study, with a response rate of 99.5 %. For all parental characteristics see Table 1. Table 2 presents the distribution of the sample according to attributes of the child, household, community and health care characteristics. Among the children, 338 (36.7 %) were in the age category of 6–11 months The mean age of children was 14.21 ± 5.27(SD) months, and 90 % of children were breast fed at a time of data collection.

**Table 1:** Parental level characteristics of children aged 06–23 months, Dangila, Northwest Ethiopia, 2014. (n = 920)

Characteristic	Frequency(n)	Percentage (%)
Parental characteristics		
Mother’s age (years)		
15–24	223	24.2
25–34	512	55.7
35–49	185	20.1
Mother’s religion		
Orthodox	758	82.4
Others^a^	162	17.6
Mother’s ethnicity		
Amhara	904	98.3
Others^b^	16	1.7
Marital status		
Currently married	827	89.9
Formerly married/not^c^	93	10.1
Mother’s education		
Cannot write and read	392	42.6
Primary education (1–8)	223	24.2
Secondary education (9–12)	219	23.9
Higher education^d^	86	9.3
Father’s education (n = 848)		
Cannot write and read	252	29.7
Primary education (1–8)	222	26.2
Secondary education (9–12)	244	28.8
Higher education^d^	130	15.3
Mother’s work		
Currently not work^e^	695	75.5
Currently working	225	24.5
Father’s work (n = 848)		
Farmer	355	41.9
Merchant	149	17.6
Government employee	197	23.2
Non government employee	17	2
Self employee	74	8.7
Labor work	56	6.6

^a^Muslim/protestant/catholic, ^b^Oromo/Tigre, ^c^single/divorced/died, ^d^college/university, ^e^housewife/student

**Table 2:** Child, household, community and health care level characteristics of children aged 6–23 months, Dangila, Northwest Ethiopia, 2014

Characteristics	Frequency (n)	Percentage (%)
Child characteristics		
Age of child in months		
06–11	338	36.7
12–17	287	31.2
18–23	295	32.1
Sex of a child		
Male	436	47.4
Female	484	52.6
Birth order of index child		
First	259	28.2
Second to fourth	553	60.1
Above fourth	108	11.7
Currently breast feed		
No	100	10.9
Yes	820	89.1
Diarrhea for the last two weeks		
No	798	86.7
Yes	122	13.3
ARI for the last two weeks		
No	814	88.5
Yes	106	11.5
Household characteristics		
No of <5 children		
One	622	67.6
Two	289	31.4
Three and above	9	1
Decision making at household		
Mothers not involved	206	22.4
Mothers involved	714	77.6
Exposure to media		
Unsatisfactory	567	61.6
Satisfactory	353	38.4
Household Wealth		
Poor	305	33.2
Middle	314	34.1
Rich	301	32.7
Sources of information on IYCF^a^		
Health professionals	703	76.4
Relatives	133	14.5
Friends	149	16.2
Family	155	16.8
Media promotion (radio/television)	171	18.6
No information	82	8.9
Community characteristics		
Residence		
Rural	520	56.5
Urban	400	43.5
Home gardening		
No	825	89.7
Yes	95	10.3
Uses of home gardening (n = 95)		
Only for sell	18	18.9
Only for household consumption	49	51.6
Both for cell and household	28	29.5
Health care characteristics		
Antenatal clinic visits		
Missing	142	15.4
1–3 times	156	17
Four and above times	622	67.6
Place of delivery		
Home	229	24.9
Institution	691	75.1
Timing of post-natal check-up		
Missing	295	32.1
Within 1–2 days	81	8.8
Within 3–6 days	161	17.5
After 7 days	383	41.6

^a^Proportion cannot be 100 % (it is based on multiple option questions)

**Table 3:** Types of food groups practiced among 6–23 months children in Dangila, Northwest Ethiopia, 2014

Food groups	Frequency(n)	^a^Percentage (%)
1. Grains, roots and tubers	738	80.2
2. Legumes and nuts	544	59.1
3. Dairy products	452	49.1
4. Flesh food	22	2.4
5. Eggs	108	11.7
6. Vitamin A rich fruits and vegetables	131	14.2
7. Other fruits and vegetables	68	7.4

^a^Proportion cannot be 100 % (it is based on multiple option questions)

### Practices of dietary diversity and meal frequency

Table 3 indicates the types of food groups practiced by study subjects. Grains, roots and tubers were eaten by 80.2% of children. The present study found that only 12.6 % of children received the recommended dietary diversity, which is lower as compared with the DHS reports of developing countries from Africa, Asia, and Latin America [10]. This low dietary diversity coverage is also similar with different studies conducted in Ethiopia (10.8 %) [9], Democratic Republic of Congo (12 %), Burkina Faso (14 %), Mali (16 %), and India (15.2 %) [10, 11]. However, it is lower than findings from Nepal (34 %), East New Delhi (33 %), Bangladesh (41.9 %), Nepal (72 %), and Sri Lanka (71 %) [12, 13, 15–17] (Fig. 1).

The difference could be due to lack of awareness about nutritional requirements for infants and young children, affordability to a food product and low purchasing power for food. This population has also different feeding habit with a tradition of cooking few verities of food for the family. Moreover, there appears to be a tendency to share food with siblings at home.

Proportion of children who received minimum meal frequency found to be 50.4 %. The practice is higher as compared to EDHS report (44.7 %) [9], Mali (25 %), Burkina Faso (31 %), Democratic Republic of Congo (30 %), Cameron (41 %) and India (42 %) [10, 11]. It is similar with studies conducted in New Delhi (49 %), Vietnam (48 %), Namibia (49 %) [10, 17]. But it is lower when compared with studies from Asia and Latin American countries like Nepal (82 %), Kathmandu (65 %), Bangladesh (81 %), Sri Lanka (88 %), and Peru (78 %) [10, 12, 13, 15, 16].

As we see the meal frequency practice is higher compared with same African countries this might be due to difference in feeding habits and had better production and purchasing power compared with others relatively. But much lower than Asian countries, this difference might be due to educational level, habit of feeding frequency, lack of knowledge about how many time solid, semisolid and soft food should be given for a child and even if had knowledge lack of affordability to enough food production and purchasing power.

### Factors affecting dietary diversity

The educational status of a mother, age of a child, birth order of index child, area of residence, home gardening and satisfactory media exposure of a mother were significantly associated with providing the minimum dietary diversity after controlling for other predictors in the model (Table 4).

**Table 4:** A bivariate and multivariate logistic regression output showing factors associated with minimum dietary diversity practice among 06 to 23 months children, Dangila, Northwest Ethiopia, 2014

	Minimum dietary diversity			
Characteristics	Inadequate	Adequate	COR (95 % CI)	AOR (95 % CI)
Mother's education				
Cannot write and read	359 (91.60)	33 (8.40)	1.00	1.00
Primary education (1–8)	199 (89.20)	24 (10.80)	1.312 (0.754,2.282)	1.539 (0.831,2.852)
Secondary education (9–12)	182 (83.10)	37 (16.90)	2.212 (1.339,3.654)	2.516 (1.284,4.929)
Higher education	64 (74.40)	22 (25.60)	3.740 (2.049,6.824)	4.230 (1.918,9.332)
Mother's work				
Currently not working	616 (88.60)	79 (11.40)	1.00	1.00
Currently working	188 (83.60)	37 (16.40)	1.535 (1.005,2.343)	1.028 (0.618,1.712)
Age of a child(months)				
06–11	315 (93.20	23 (6.80)	1.00	1.00
12–17	248 (86.40)	39 (13.60)	2.154 (1.253,3.701)	2.047 (1.172,3.575)
18–23	241 (81.70)	54 (18.30)	3.069 (1.832,5.141)	2.889 (1.693,4.931)
Order of index child				
First	233 (90.00)	26 (10.00)	1.00	1.00
Second to fourth	479 (86.6)	74 (13.4)	1.384 (0.862,2.223)	2.077 (1.235,3.494)
Above fourth	92 (85.20)	16 (14.80)	1.559 (0.799,3.039)	2.758 (1.258,6.046)
ARI status				
No	706 (86.3)	108 (13.7)	1.00	1.00
Yes	98 (92.5)	8 (7.5)	0.534 (0.252,1.128)	0.571 (0.264,1.235)
Residence				
Rural	448 (86.20)	72 (13.80)	1.00	1.00
Urban	356 (89.00)	44 (21.00)	0.769 (0.515,1.147)	2.094 (1.117,3.926)
Home gardening				
No	728 (88.20)	97 (11.80)	1.00	1.00
Yes	76 (80.00)	19 (20.00)	1.876 (1.087,3.238)	2.031 (1.093,3.775)
Decision making at household				
Mothers not involved	190 (92.20)	16 (7.80)	1.00	1.00
Mothers involved	614 (86.00)	100 (14.00)	1.934 (1.113,3.360)	0.913 (0.482,1.731)
Media exposure				
Unsatisfactory	521 (91.90)	46 (8.10)	1.00	1.00
Satisfactory	283 (80.20)	70 (19.80)	2.802 (1.879,4.176)	2.738 (1.517,4.943)

The study found that children born from mothers who were well educated and had a secondary level education [AOR 2.52; 95 % CI (1.28, 4.93)] or higher education [AOR 4.23; 95 % CI (1.92, 9.33)] had greater odds of feeding diversified foods. A recent study done on comparison of five Asian countries on infant feeding reports that mother’s education is a significant determinant of appropriate diversified infant feeding [18, 19]. Sri Lanka had the highest proportion of children meeting the infant feeding guidelines for diversity; and this is linked to the higher education status of mothers and overall literacy [12]. Similar positive impact of education on diverse feeding practices is also reported in a previous studies in Nepal, Bangladesh, Indonesia, India including Ethiopia [9, 19]. This could be educated mothers are more likely to have information (media exposure), understand the education message, more likely to be engaged in the paid work and might have received lessons on child feeding in the curricula at school.

Another most important factor significantly associated with minimum dietary diversity was age of a child. Children aged 12–17 and 18–23 months had about two times higher odds [(AOR 2.05; 95 % CI (1.17–3.58) and (AOR 2.89; 95 % CI (1.69, 4.93)] of having minimum dietary diversity compared to children aged 6–11 months. This study is in line with studies conducted in Ethiopia, Indonesia, Nepal, and Sri Lanka [9, 12, 15, 19]. This indicated the relationship between different food groups by age group which implies that food groups decrease as the child age decreases. This might be due to late introduction of complimentary feeding and when they start complimentary feeding on time; they included only milk or cereal products like gruel. Other possibility could be mothers may perceive that younger the child, the poor ability of child’s intestine to digest solid, semisolid and soft foods. Besides, mothers may assume introducing a bulk of food would lead them to develop infections [20].

It was found that birth order of index child had significant association with dietary diversity. Children born in the second to fourth order [AOR 2.08; 95 % CI (1.24, 3.49)] and above fourth order [AOR 2.76; 95 % CI (1.26, 6.05)], respectively, had about two and three times higher odds of having the minimum dietary diversity compared with children who were born in first order. This result is contradictory to that of previous studies conducted on 2011 EDHS analysis [9]. This difference might be due to study area, sample size and time horizon. This study is conducted in more or less homogenous community with limited sample size; but the EDHS study included large population from different ethnic and regions with various culture, beliefs, and traditions such as a tendency to prioritizing the first child from his/her younger siblings. Another possible reason for this difference may be that as a mother’s parity increase, she gets experience on how to prepare and feed diversified diet to her child.

This study also indicated that children born from mothers who lived in urban areas were reported higher practice of minimum dietary diversity [AOR 2.09; 95 % CI (1.12, 3.93)] as compared to those children born from mothers who lived in rural areas. This is similar to study conducted in Indonesia [21]. The low practice of diet diversity in rural region may be due to lake of awareness regarding importance of dietary diversity in rural community compared to urban community, which has access to mass media. Another difference may be traditional beliefs and practices. During introducing complimentary food to infants in rural community, infants may develop diarrhea due to poor hygienic condition, but mothers could associate this problem with taking different food items and eventually she might not permit the child to taste unfamiliar foods. Those children with parents possessing home gardening had two times [AOR 2.03; 95 % CI (1.09–3.78)] higher odds of having the minimum dietary diversity as compared to children whose parents did not. This could indicate that parents with home gardening would grow vegetables and fruits and then they child would get additional options in his/her diet. This finding is supported by a study conducted in Southern Ethiopia [22].

Children whose mothers who had been exposed to media had a higher odds of having diversified diet [AOR 2.74; 95 % CI (1.52, 4.94)] than those children of mothers who had not been exposed to media. This is similar studies shown in Ethiopia, India, and Sri Lanka [9, 11, 12]. This might be pointing to the influence of the media on infant and young child feeding practices. This could have happened due to the promotions of child nutrition related media advertisement in national radio and television.

### Factors affecting minimum meal frequency practice

Age of a child, birth order of an index child, involvement of mother in decision making in the household, satisfactory media exposure of a mother and time of postnatal care visit were significantly associated with the recommended minimum meal frequency after controlling for other predictors in the model (Table 5).

**Table 5:** A bivariate and multivariate logistic regression output showing factors associated with minimum meal frequency practice in 06 to 23 months children, Dangila, Northwest Ethiopia, 2014

Characteristics	Minimum meal frequency			AOR (95 % CI)
	Inadequate	Adequate	COR (95 % CI)	
Mother's education				
Cannot write and read	214 (54.60)	178 (45.40)	1.00	1.00
Primary education (1–8)	123 (55.20)	100 (44.80)	0.977 (0.702,1.36)	0.912 (0.612,1.359)
Secondary education (9 12)	85 (38.80)	134 (61.20)	1.895 (1.353,2.654)	1.347 (0.832,2.181)
Higher education	34 (39.50)	52 (60.50)	1.839 (1.143,2.959)	1.022 (0.531,1.966)
Mother’s work				
Currently not work	358 (51.50)	337 (48.50)	1.00	1.00
Currently working	98 (43.60)	127 (56.40)	1.377 (1.017,1.863)	1.180 (0.837,1.664)
Age of a child(months)				
06–11	237 (70.10)	101 (29.90)	1.00	1.00
12–17	125 (43.60)	162 (56.40)	3.041 (2.187,4.229)	3.025 (2.141,4.274)
18–23	94 (31.90)	201 (68.10)	5.018 (3.579,7.035)	5.028 (3.524,7.175)
Birth Order of index child				
First	135 (52.10)	124 (47.90)	1.00	1.00
Second to fourth	270 (48.80)	283 (51.20)	1.141 (0.849,1.533)	1.580 (1.133,2.205)
Above fourth	51 (47.20)	57 (52.80)	1.217 (0.776,1.908)	1.778 (1.068,2.958)
Breast feeding status				
No	41 (41.00)	59 (59.00)	1.00	1.00
Yes	415 (50.60)	405 (49.40)	0.671 (0.445,1034)	0.895 (0.550,1.454)
Decision making at household				
Mothers not involved	127 (61.70)	79 (38.30)	1.00	1.00
Mothers involved	329 (46.10)	385 (53.90)	1.881 (1.370,2.583)	1.512 (1.053,2.170)
Media exposure				
Unsatisfactory	335 (59.10)	232 (40.90)	1	1.00
Satisfactory	121 (34.30)	232 (65.70)	2.769 (2.100,3.65)	2.620 (1.901,3.611)
Residence				
Rural	240 (46.20)	280 (53.80)	1.00	1.00
Urban	216 (54.00)	184 (46.00)	1.37 (1.054,1.779)	1.243 (0.849,1.821)
Home gardening				
No	418 (50.70)	407 (49.30)	1.00	1.00
Yes	38 (40.00)	57 (60.00)	0.050 (1.000,2.374)	1.412 (0.878,2.273)
Place of birth				
Home	240 (46.20)	280 (53.80)	1.00	1.00
Institution	216 (54.00)	184 (46.00)	1.277 (0.946,1.723)	1.045 (0.689,1.583)
Time of post natal care visit				
Missing	158 (53.60)	137 (46.40)	1.00	1.00
Within 1–2 days	22 (27.20)	59 (72.80)	3.093 (1.802, 5.310)	2.295 (1.269, 4.150)
Within 3–6 days	78 (48.40)	83 (51.60)	1.227 (0.835,1.803)	0.860 (0.553,1.337)
After 7 days	198 (51.70)	185 (48.30)	1.078 (0.795,1.461)	0.848 (0.598,1.189)

*AOR* Adjusted Odd Ratio, *COR* Crude Odd Ratio, *CI* Confidence Interval

The study showed that children with age group of 12–17 months [AOR 3.03; 95 % CI (2.14,4.27)] and 18–23 months [AOR 5.03; 95 % CI (3.52,7.18)] had higher odds of recieving the minimum frequency in their daily meal compared to children age group between 6-11months. This study also supported by studies conducted in Ethiopia, India, and Seri Lanka [9, 11, 12]. This might be occurred due to the fact that for the infants during 6–11 months, mothers did not introduce semi solid and soft food; they are simply fed on animal or canned milk along with breast milk. Unfortunately, however, the definition of minimum meal frequency, did not not consider breast milk while calculating minimum meal frequency for breast feed infants.

The study found that Children born in the second to fourth order [AOR 1.58; 95 % CI (1.13, 2.21)] and above fourth order [AOR 1.78; 95 % CI (1.07, 2.96)] were more likely to met the minimum meal frequency as compared with children who were born first order. This difference could be due to mothers who give birth for first time may have less knowledge than those of multi parity mothers. And also as mother’s parity increased mothers become experienced how to feed children frequently.

Involvement of mothers in household decision making found to be significantly associated with minimum meal frequency. Children from mothers involved in decision making in the house hold were 1.5 times[AOR 1.51; 95 % CI (1.05–2.17)] more likely to provide the recommended meal frequency as compared to the children from the mothers not involved in decision making in the household. This study also in line with study conducted in India [11]. The possible explanation for this difference may be, most of the time the responsibility of child feeding is on the shoulders of mother even if the source is from husbands in Ethiopian context [20]. So participation of mothers with their household issues, they can access household resources easily and contribute that mothers can fed the children more frequently.

Children born from mothers who were exposed to media, *i.e.,* watched television, listened to radio and read newspapers or magazines every day or at least once a week has more likely to meet minimum meal frequency [AOR 2.62; 95 % CI (1.90–3.61)] than those children born from mothers who watched television, listened to radio and read newspapers or magazines less than once a week or not at all. This study is similar with other studies conducted in Ethiopia, Nepal, Seri Lanka and India [9, 11, 12, 14]. The reason behind for this could be currently there is a media promotion using radio and television that promote and show practice of IYCF. This may reflect broadly the power of mass media for improvement meal frequency practice.

Mothers who had attended PNC within 1–2 day after delivery [AOR 2.30; 95 % CI (1.27–4.15)] were more likely to provide recommended meal frequency than mothers who had no PNC visit. Nutritional counseling for mothers about frequent feeding during PNC is important continuum and Mothers who have attended PNC visits may be more informed, have greater access to services and may be from a well off family, and thus more likely to be able to afford and provide of foods more frequently to their children.

The study is not free of recall bias and social desirability bias. It may not also accurately reflect childrens’ past feeding experience since it considers only 24-hour feed. This study does not take account of the quality and amount of food provided.

## Conclusion

Infant and young children aged between 06–23 months receiving minimum dietary diversity score and minimum meal frequency is low compared with other countries.

Age of a child, birth order of index child and media exposure of a mother consistently associated both minimum dietary diversity and meal frequency practices. In addition, education level of a mother, residence and home gardening has significant association with minimum dietary diversity while mother’s involvement in household decision making and postnatal visit have significant association with minimum meal frequency. Promoting women empowerment, home gardening and nutrition education is highly recommended to improve infant and young child feeding practice.

## Abbreviations

ANC: Antenatal Care
DHS: Demographic and Health Survey
EDHS: Ethiopian Demographic and Health Survey
IYCF: Infant and Young Child Feeding
MDD: Minimum Dietary Diversity
MDG: Millennium Development Goals
MMF: Minimum Meal Frequency
PNC: Postnatal Care
WHO: World Health Organization
